# Exponential consensus ranking improves the outcome in docking and receptor ensemble docking

**DOI:** 10.1038/s41598-019-41594-3

**Published:** 2019-03-26

**Authors:** Karen Palacio-Rodríguez, Isaias Lans, Claudio N. Cavasotto, Pilar Cossio

**Affiliations:** 1 Biophysics of Tropical Diseases Max Planck Tandem Group, University of Antioquia, Medellín, Colombia; 2 Computational Drug Design and Drug Discovery Informatics Laboratory, Translational Medicine Research Institute (IIMT), CONICET-Universidad Austral, Pilar-Derqui, Buenos Aires Argentina; 3 Facultad de Ciencias Biomédicas, Universidad Austral, Pilar-Derqui, Buenos Aires Argentina; 4 Facultad de Ingeniería, Universidad Austral, Pilar-Derqui, Buenos Aires Argentina; 5 Department of Theoretical Biophysics, Max Planck Institute of Biophysics, 60438 Frankfurt am Main, Germany

## Abstract

Consensus-scoring methods are commonly used with molecular docking in virtual screening campaigns to filter potential ligands for a protein target. Traditional consensus methods combine results from different docking programs by averaging the score or rank of each molecule obtained from individual programs. Unfortunately, these methods fail if one of the docking programs has poor performance, which is likely to occur due to training-set dependencies and scoring-function parameterization. In this work, we introduce a novel consensus method that overcomes these limitations. We combine the results from individual docking programs using a sum of exponential distributions as a function of the molecule rank for each program. We test the method over several benchmark systems using individual and ensembles of target structures from diverse protein families with challenging decoy/ligand datasets. The results demonstrate that the novel method outperforms the best traditional consensus strategies over a wide range of systems. Moreover, because the novel method is based on the rank rather than the score, it is independent of the score units, scales and offsets, which can hinder the combination of results from different structures or programs. Our method is simple and robust, providing a theoretical basis not only for molecular docking but also for any consensus strategy in general.

## Introduction

Experimental methods for drug discovery involve high-throughput screening techniques, in which large numbers of compounds are experimentally tested and their activity is evaluated towards a biological target^1^. However, these procedures involve large amounts of resources and time. Computer-aided methods have emerged as a way to decrease the time and economic costs of the experimental trials by evaluating large datasets of molecules in virtual screening campaigns^2–5^. With these methods it is possible to filter compounds that are potentially active towards a protein target from large datasets. The impact of these *in silico* approaches for the discovery of new drugs has been widely documented^3,5–8^.

Molecular docking methods are commonly used in virtual screening campaigns of large chemical libraries^2,9–12^. These methods aim to find the most favourable position, orientation and conformation of each molecule upon binding to a protein target^13,14^, assigning a docking score to each molecule, which is an estimation of the likelihood of binding^15^. These high-throughput docking calculations are computationally efficient because the conformational space of the ligand is small (compared to that of the target-ligand complex) and the scoring functions are fast^16^. Thus, with molecular docking, it is possible to screen and rank molecules from large datasets.

Despite the success of protein-ligand docking in many virtual screening campaigns, there are several limitations. For example, the flexibility of the protein target is usually not completely taken into account^17–23^. To overcome this challenge, some methodologies use multiple reference target structures^24–29^ and merging and shrinking procedures^22,30–33^. Another limitation is that considerable prior knowledge of the biological system is needed, for example, for choosing the correct active site, knowing the protonation state of its amino acids, or defining the type of activity that is being searched for in the ligands^16^. In addition, the enrichment of the hit-list with actual ligands critically depends on the quality of the scoring functions^34^.

**Figure 1:**
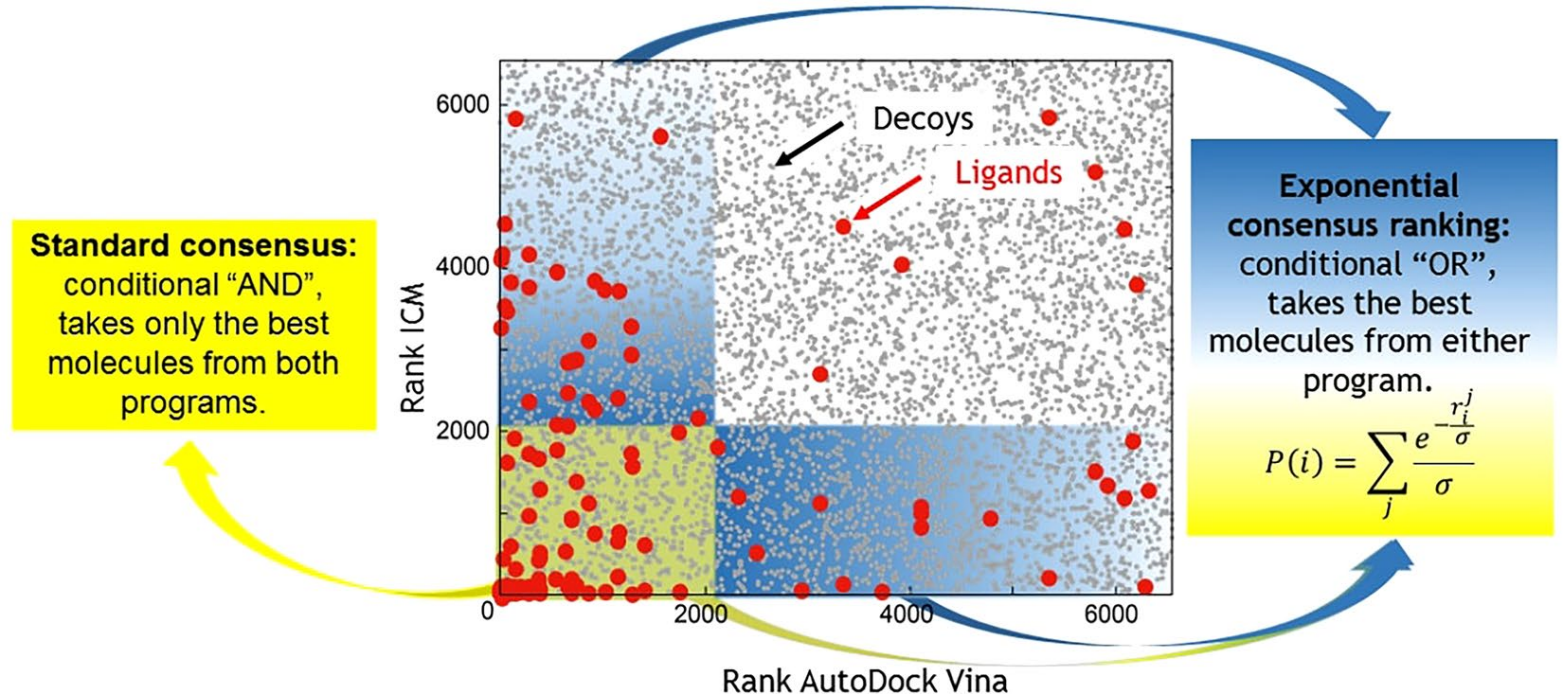
Example of the correlation between the results of two docking programs. For estrogen receptor alpha (ESR1; structure 3ERT), each point corresponds to the rank of a molecule for AutoDock Vina versus the rank of the same molecule in ICM (see Methods). Red and grey circles are ligands and decoys, respectively. There is a poor correlation between the results of docking programs, and some ligands can be ranked well by one program but poorly by another (red circles in blue region). Traditional consensus approaches only take the best molecules for all programs (yellow region), acting as a conditional “*and*”, whereas the novel exponential consensus ranking (ECR) strategy takes the best molecules from either program, acting as a conditional “*or*” (taking both the yellow and blue regions).

It has been found that combining results from different docking programs (*i*.*e*., consensus scoring) to obtain a final rank or score for each molecule leads to a higher success rate in virtual screening processes^12,34–39^. Traditional consensus methods select molecules that have the best performances for all scoring functions (*i*.*e*., the intersection between the results of the docking programs^35^; *e*.*g*., Fig. 1 yellow region). Alternative rank-based consensus strategies, such as ‘rank-by-rank’ and ‘rank-by-vote’^40^, or score-based strategies, such as ‘average of auto-scaled scores’^36^, ‘Z-score’^41^, and ‘rank-by-number’^40^, have become popular in recent years^38,41,42^ (see the Methods for a detailed description). Although these consensus methodologies have been shown to produce better results than individual scoring functions/docking programs, their implementation may be subject to biases and errors within data management^40^, as we show later. Consensus docking methodologies have also been used to successfully obtain the best poses of molecules in the binding pocket from multiple docking programs^43–45^. However, this work focuses on developing a method to extract only the best rank for each molecule from a consensus perspective.

Here, we propose a new consensus scoring function that combines results from several docking programs using an exponential distribution for each individual rank. We tested the novel method over a wide range of datasets and found that it performed as good as or better than the top consensus strategies. Moreover, the results of the novel method are parameter-independent over a wide range. This paper is organized as follows. First, we describe the mathematical theory of the exponential consensus method. In the Results section, we show the performance of the individual docking programs in comparison to consensus strategies over different datasets, and then, we assess the performance of the consensus metrics in receptor ensemble docking. Finally, the conclusions are presented.

## Theory

There are three types of docking scoring functions: force-field based, empirical and knowledge based^46^. The performances of these scoring functions depend on the training sets used, empirical assumptions, and parameterization protocol. It has been shown that scoring functions poorly correlate with binding free energies^47^; moreover, the correlation of the results between different docking programs is also poor. As an example, in Fig. 1, we show the correlation between rankings using the ICM program^48^ versus AutoDock Vina^49^ for estrogen receptor alpha (ESR1; structure 3ERT). Although both programs are designed to distinguish between ligands and decoys (red and grey circles, respectively, in Fig. 1), we find that while one program can predict a good rank for a molecule, the other program might not (red circles in blue regions Fig. 1).

Traditional consensus approaches select the intersection of the best results between docking programs, *i*.*e*., the molecules that perform well in all programs (shown by the yellow region of Fig. 1). These methods discard molecules that perform poorly in at least one program (*e*.*g*., blue regions in Fig. 1). Thus, if the results from all programs were highly correlated, then traditional consensus approaches would work well. However, because the docking programs are not optimal and have highly uncorrelated outcomes, it is necessary to propose alternative consensus strategies that will be able to select molecules that perform well in some programs but poorly in others (acting qualitatively as a conditional “*or*”).

For this purpose, we propose *Exponential Consensus Ranking* (ECR), a strategy to combine the results from several scoring functions/docking programs using an exponential distribution for each individual rank. We assign an exponential score $p({r}_{i}^{j})$ to each molecule (*i*) for each scoring function (*j*) using the rank of the molecule (${r}_{i}^{j}$) given by each individual docking program,

1 $$p({r}_{i}^{j})=\frac{1}{\sigma }\,\exp \,(\,-\,\frac{{r}_{i}^{j}}{\sigma }),$$

where *σ* is the expected value of the exponential distribution. This parameter establishes the number of molecules for each scoring function that will be considered, *i*.*e*., the threshold of the dataset to be taken into account for the consensus. The final score of each molecule *i* is defined as the sum of the exponential score for all of the scoring functions *j*

2 $$P(i)=\sum _{j}\,p({r}_{i}^{j})=\frac{1}{\sigma }\,\sum _{j}\,\exp \,(\,-\,\frac{{r}_{i}^{j}}{\sigma }).$$

We note that the expected value *σ* can be different for each docking program. However, for the sake of simplicity, we kept this value constant. Importantly, we found that the ECR results were almost independent of *σ* (see the Results and Supplementary Information).

Since the score of each molecule *P*(*i*) corresponds to the summation of the ECR scores from all of the scoring functions, ECR assigns a higher score for molecules that are well ranked by several programs. However, by summing over step-like or sigmoid distributions, such as an exponential or a Gaussian function, we are able to select molecules that rank well for *any* of the docking programs, but it is not mandatory that the molecules rank well for *all* of the docking programs. The advantage of summing over sigmoid-like distributions is shown in the Supplementary Information, Table S1, for a simple case for which one program gives a very poor rank to a molecule while all the rest rank it highly. In summary, ECR acts qualitatively as a conditional “*or*”, *e*.*g*., by taking the molecules that fall both in the yellow and blue regions in Fig. 1 into account.

As an additional point, we also note that ECR can give the same score to many poor-performing molecules (*e*.*g*., the worst molecules can all have a score of 0), which leads to complications for correctly determining the rank of molecules that have the same score. Therefore, it is necessary to shuffle molecules that have the same score several times and calculate the average and standard deviation of the performance metrics, such as the enrichment factors. A similar issue but for all molecules (not only poor-performing ones) occurs for the RbV metric with the number of votes (see the Methods).

## Results and Discussion

### Consensus ranking improves enrichment using a single target structure

The performance of an array of different docking programs has been widely evaluated on several systems^47,50,51^. It has been found that the effectiveness of each program is system-dependent, mainly because the search algorithms used to find the correct poses and scoring functions depend on the training sets and parameterization protocols. In this work, we use six docking programs: AutoDock^52^, ICM^48^, LeDock^53^, rDock^54^, AutoDock Vina^49^ and Smina^55^. All these programs have search algorithms and scoring functions based on different approximations and parameters (see the Methods). To mimic a scenario in which a docking program has poor results, we introduce a scoring function that assigns random scores to each molecule according to a normal distribution. This Random Scoring Function (RSF) simulates the results of a docking program that fails to properly distinguish between ligands and decoys (*cf*. the ROC-AUC in Supplementary Table S2).

We analyse the results of the docking programs over four diverse benchmark systems: cyclin-dependent kinase 2 (CDK2), estrogen receptor *α* (ESR1), *β*_2_ adrenergic receptor (ADRB2), and carbonic anhydrase 2 (CAH2) with two target crystal structures each (see the Methods). In Table 1, we show the enrichment factor at 2% (EF2) (see the Methods) for each system and structure, and for each individual docking program (EF1, EF5, enrichment plots (EP) and ROC-AUC are presented in Supplementary Tables S2–S4 and Supplementary Fig. S1). In agreement with the literature, the performance of the docking programs is system-dependent and, in some cases, structure-dependent for the same system^16,39,47,50^. On average, ICM presented the best performance, followed by rDock, while AutoDock Vina and Smina presented worse performances. However, for the CAH2 system ICM had one of the worst performances. LeDock presented the best results for CDK2 but poor results for ESR1 and ADRB2, possibly because it was parameterized with a set of protein kinases^56^. No program presented the best performance over all of the structures and systems evaluated; moreover, in some cases, docking programs, such as AutoDock Vina, presented a similar performance to that of the RSF (see Table 1 for the CDK2 structure 4KD1 and ROC-AUC in Supplementary Table S2). Overall, these results highlight potential problems in over-fitting and training-set dependencies.

**Table 1:** EF2 for all individual docking programs and consensus strategies using individual structures.

System	CDK2		ESR1		ADRB2		CAH2	
Structure	4KD1	1FVV	1XP9	3ERT	3PDS	4LDO	1BCD	4PQ7
AutoDock	3.0	13.0	14.6	13.8	3.6	5.8	0.7	2.2
ICM	6.0	14.0	18.9	18.9	11.7	17.5	1.5	3.2
LeDock	10.0	14.0	6.7	9.8	2.2	3.6	8.1	7.1
rDock	4.0	16.0	16.5	19.7	6.6	6.8	5.1	8.8
Smina	5.0	6.0	15.4	15.0	4.4	1.2	3.4	5.6
AutoDock Vina	2.0	7.0	9.8	9.5	4.6	2.2	3.2	2.0
RSF	2.0	2.0	0.8	0.4	0.0	1.0	1.2	0.7
AASS	7.0	20.0	11.4	10.2	3.2	5.1	8.8	6.8
RbN	7.0	13.0	18.9	17.3	8.0	15.8	5.6	6.3
Z-score	6.0	19.0	18.1	20.5	9.2	10.0	10.5	9.8
RbR	7.0	17.0	17.7	18.5	9.0	10.0	9.5	8.1
RbV	6.8	19.1	19.9	21.1	9.2	9.5	8.3	9.3
ECR	6.0	21.0	21.7	22.8	11.9	10.4	8.3	9.0

RbV and ECR strategies were calculated for a threshold equal to the 5% of the dataset.

**Figure 2:**
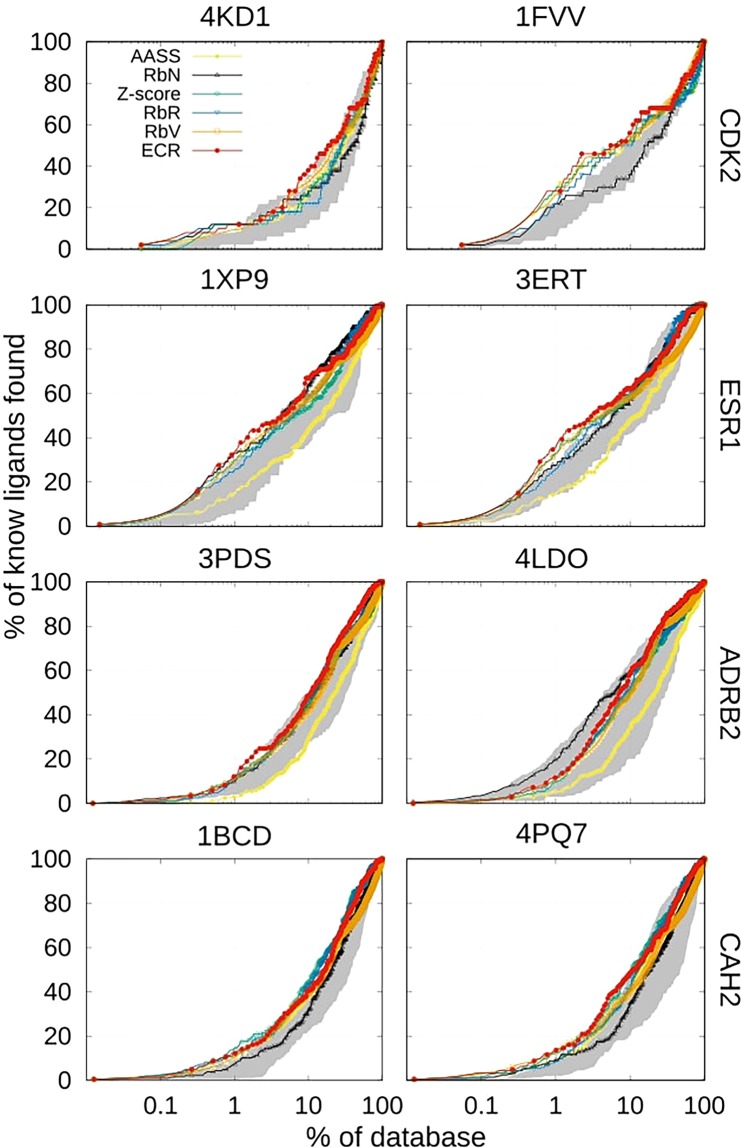
Enrichment plots for consensus strategies using the results obtained from each individual program using individual structures. The shaded area corresponds to the region between individual docking programs that presented the best and worst performance for each system. RbV and ECR were calculated using a threshold-parameter of 5% of the dataset.

In Table 1, we also compare state-of-the-art consensus strategies [average of auto-scaled scores (AASS), rank-by-number (RbN), Z-score, rank-by-rank (RbR) and rank-by-vote (RbV), (see the Methods)] with ECR over each target structure for all of the benchmark systems. In Fig. 2, we present the EP (for the definition, see the Methods) for the consensus strategies over the benchmark systems and individual structures. The shaded regions correspond to the area between the EPs of the best and worst performances of the individual docking programs (the individual results of all the programs are presented in Supplementary Fig. S1).

As previously shown^38,42,57^, it is observed that consensus strategies improve the results obtained from individual docking programs when screening up to the top-ranked 10% of the dataset (as is usually desired). However, the performance of the score-based consensus strategies, such as RbN and AASS, is system-dependent. For example, RbN has the best performance for ADRB2 (structure 4LDO); but the worst performance for CDK2 (structure 1FVV) because RbN is biased toward programs with more negative scores (see the score distributions in Supplementary Fig. S3), which are ICM and rDock.

The system dependence of consensus strategies can be proven by generating the same table without taking the ICM results into account (see Supplementary Table S5), leading to marked deterioration of the results of RbN. On the other hand, AASS has poor performance for the ESR1, ADRB2 and CAH2 systems, despite the fact that it is not affected by the scoring scales of each program.

Rank-based consensus strategies avoid problems that occur when combining scores with different scales or offsets from individual docking programs, making them less subject to bias. This problem avoidance is demonstrated by observing that the results of the RbR, RbV and ECR consensus strategies are consistent over all of the systems. However, it is important to note that the RbV and ECR consensus strategies depend on an additional parameter that considers the percentage of the dataset that will be taken into account during the consensus protocol (see the Theory and Methods sections). The dependence of these two strategies on the threshold parameter is shown in Supplementary Table S6, where it is evident that RbV depends, to a large extent, on the threshold of the dataset that is taken into consideration in the consensus, while ECR does not.

In addition, because the score of each molecule in RbV is the number of votes received at a certain threshold (see the Methods), it is possible that a large number of molecules have the same number of votes, which can lead to large uncertainties in determining the actual rank of each molecule. A similar issue could occur in ECR, but only for molecules that have been poorly ranked by all the programs, *e*.*g*., those that have a score near zero. It is an advantage for ECR over the RbV, when only the poor-ranking molecules have uncertainties, instead of all the whole set. To estimate the error in the enrichment factors and enrichment plots due to the uncertainty in ranking molecules that have the same score, the ranking lists for these molecules were randomly shuffled 20 times. The mean and standard deviation was estimated for the EFs and EPs for the RbV and ECR strategies for molecules that had the same score. In Supplementary Fig. S4, it is observed that the RbV results are subject to large error due to the shuffling, while the ECR results are not. These results show several advantages of the ECR: (*i*) the stability of the results with respect to the system and structure, (*ii*) the ability of ECR to overcome problems stemming from different score scales and offsets, (*iii*) the independence of the results of ECR from the *σ* parameter, and (*iv*) the small uncertainties of ECR after shuffling molecules with the same score.

The results presented in Table 1 and Fig. 2 show that the performance of most of the strategies depends not only on the system considered but also on the target structure. In the case of CDK2, the 1FVV structure presents considerably better results than the 4KD1 structure, although both structures were prepared in the same way. Thus, minimal differences within the active site can affect the docking and consensus results, as has been shown. This result highlights the relevance of accounting for target flexibility in molecular docking using procedures such as the merging and shrinking strategy, as described below.

### Consensus ranking also improves receptor ensemble docking

The merging and shrinking (MS) procedure (see the Methods) is commonly used to combine docking results from different structures of a receptor and has been widely applied to a variety of systems^22,26,30–33^. In this procedure, the best rank or score of an ensemble of structures is kept for each molecule. The EFs at 2% for the benchmark systems, using MS with the rank or the score, are presented for each individual docking program in Table 2 (first seven rows). We find that, in most cases, by applying this procedure, the EFs improve or remain similar to those presented by the best individual structure (see Supplementary Table S11) using either the rank or the score. In a few cases, the results are significantly worse than for the best individual structure (*e*.*g*., for rDock in CDK2 and Smina in ADRB2). Importantly, as has been previously shown in other systems^22,58^, the results are not as poor as those corresponding to the worst structure of each system. For example, in the case of CDK2, if the 4KD1 structure had been chosen at random, then the results would have been much worse than those obtained using MS with the two structures. Since, for a system of interest, it is difficult to know *a priori* what the best structure is, the use of the MS procedure provides a robust strategy to obtain the most valuable results. The results also indicate that the performance of the individual docking programs in receptor ensemble docking is system dependent. For example, LeDock performs best on the CDK2 system but severely under-performs on the ESR1 and ADRB2 systems, highlighting the necessity of applying consensus strategies.

**Table 2:** EF2 for MS strategy using each individual program and consensus strategies.

System	CDK2		ESR1		ADRB2		CAH2	
Merging and Shrinking	Score	Rank	Score	Rank	Score	Rank	Score	Rank
AutoDock	12.0	11.0	14.6	14.6	5.3	4.9	1.2	1.2
ICM	12.0	14.0	20.5	20.9	18.0	18.0	2.0	2.2
LeDock	15.0	15.0	8.7	9.1	2.2	2.7	7.8	7.6
rDock	11.0	11.0	17.7	19.3	8.0	8.0	7.3	8.1
Smina	6.0	7.0	15.8	15.4	1.9	2.4	4.4	4.4
AutoDock Vina	7.0	4.0	9.8	11.0	3.4	3.4	2.0	1.4
RSF	2.0	1.0	1.2	1.2	0.5	0.5	1.5	1.5
AASS	18.0		17.3		4.3		9.0	
RbN	13.0		22.1		12.9		7.8	
Z-score	18.0		22.1		9.7		10.2	
RbR		13.0		19.3		8.5		9.5
RbV		17.2		22.3		10.2		9.0
ECR		16.0		23.2		12.9		10.0

The MS procedure uses the best score or the best rank depending on the consensus strategy employed. RbV and ECR strategies were calculated for a threshold-parameter equal to 5% of the dataset.

One must be careful when applying consensus strategies with the MS procedure. As shown in the Methods section, there are two sets of consensus strategies: score-based (AASS, RbN, Z-score) and ranked-based (RbR, RbV, ECR). In score-based approaches, the units, scale or offset might be different for each program and for each individual structure as well. For example, for two structures and a single docking program, it is possible that the score distributions will not overlap due to an offset difference. When performing MS using the score, only the lowest scoring molecules for the structure with the lowest offset will be used, which may be problematic because the structure with the lowest offset may not be the one with the best performance. On the other hand, when using the rank in the MS procedure, the best molecules for each structure are considered (regardless of the offset), which removes the uncertainties of the score offset and scale between structures. In agreement with these observations, score-based consensus strategies are only applied when performing MS using the score, while rank-based consensus strategies are only applied when performing MS using the rank.

In Table 2, the results of the score-based and rank-based consensus strategies are shown, including the novel ECR strategy with rank-based MS. For the CAH2 system (a case in which most docking programs perform slightly better than random), the consensus and MS strategies improve the outcome. For the ESR1 and ADRB2 systems, MS also improves the results for the consensus strategies compared to the individual structures, probably because there was not a large difference between the results of the considered structures. However, due to the different results obtained for the two structures of CDK2, MS slightly decreases the performance of the consensus strategies compared to the best structure (1FVV). Despite this result, we can conclude that MS generates more reliable results when random target structures are chosen. EF1, EF5, EPs and ROC-AUC for the individual programs and consensus scoring when applying the MS procedure are presented in Supplementary Tables S7–S9 and Supplementary Fig. S2.

Again, the dependence of the results on the system used for some of the consensus strategies is evident. For example, RbN presents the best performance for ADRB2 but the worst performance for CDK2, because for ADRB2 the ICM program presents, by far, the best results. For this particular case, as previously stated, RbN is favoured because ICM is also the program that assigns the lowest scores. However, if the ICM program is not taken into account, the RbN results worsen from 12.9 to 5.1 at EF2 for ADRB2 (see Supplementary Table S10), similar to the results obtained for a single structure. This result reaffirms the fact that consensus strategies can be biased by the individual results of some programs.

**Figure 3:**
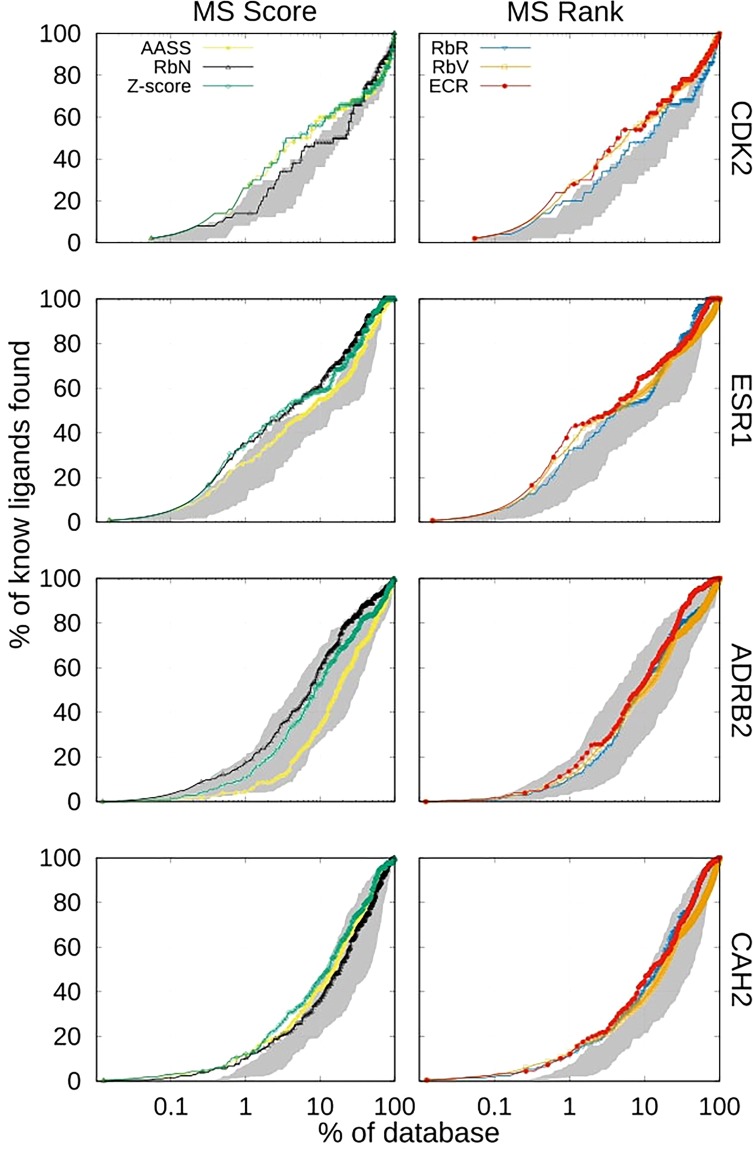
EPs for consensus strategies after applying MS procedure with score (left) and rank (right). The shaded area corresponds to the region of the best and worst performance for individual docking programs after applying MS for each system. RbV and ECR were calculated for a threshold-parameter of 5% of the dataset.

In Fig. 3, we show the EPs for MS rank-based and score-based consensus strategies. The dependency on a particular system for some of these strategies can more clearly be observed. However, rank-based consensus strategies are more stable than score-based ones, with ECR being the rank-based consensus strategy that presents the best performance overall.

### Analysis of the performance of ECR

In Table 3, we present the overall performance of each consensus strategy. To do so, we define a performance metric that counts how many times each consensus scoring strategy had the best EF for individual structures and the MS approach for all of the systems and structures studied. The performance metric for each strategy for the EFs at 1%, 2% and 5% and total are presented. In the cases for which two consensus strategies displayed the highest EFs, one point was given to each. The maximum number of points for individual results that a consensus strategy can reach is eight (since eight individual structures were evaluated), and for the MS results, the maximum score is three for either rank-based or score-based strategies. For example, for CDK2 and EF2 (see Table 2), one point is given to RbV (the best MS ranked-based strategy) and one point is given to both the AASS and the Z-score (the best MS score-based strategies). In this way, the success rate of each consensus strategy can be measured. The results presented in Table 3 show that ECR presents the best performance and demonstrate that ECR is the least dependent on the docking programs, structures or systems.

**Table 3:** Summary of the success of the different consensus strategies.

Consensus strategy	AASS	RbN	Z-score	RbR	RbV	ECR
EF1	3	4	6	1	2	7
EF2	2	4	5	1	1	7
EF5	1	4	5	0	1	9
Total	6	12	16	2	4	23

For each EF, a score of one (1) is given to the consensus strategy that presents the highest EF and zero (0) otherwise. In the case two or more consensus strategies have the same and highest EF, each strategy receives a point. The maximum number of points that can be obtained for a consensus method is nine (six for the individual structures and three for the MS).

**Figure 4:**
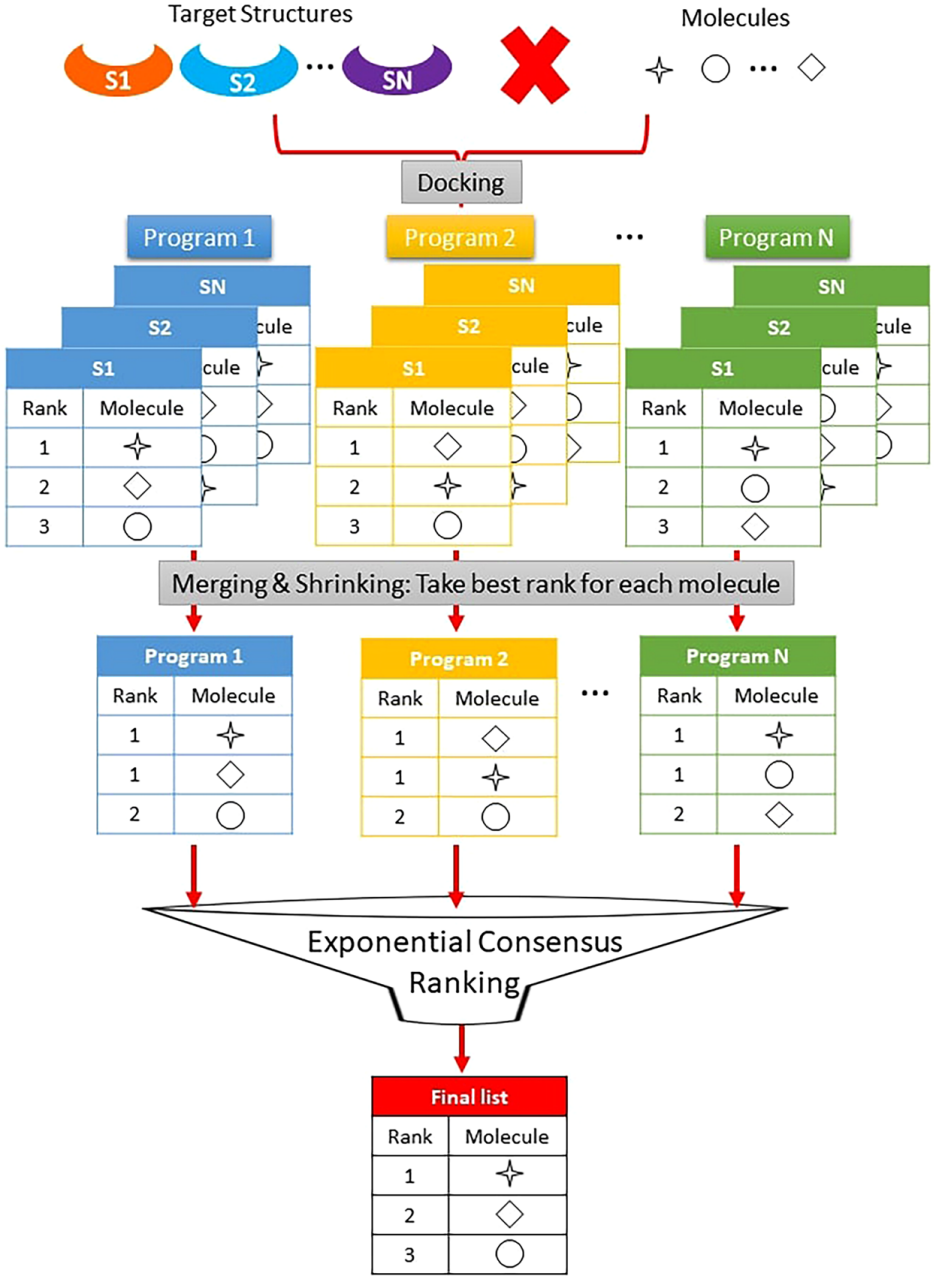
Proposed work flow to obtain a reliable and good performance using MS and ECR strategies. First, perform the virtual screening using different structures of the receptor and different docking programs/scoring functions. Then, for each program, apply the MS procedure maintaining the best rank of each molecule. Finally, apply ECR as consensus scoring method.

Taking these results into account, we present the protocol illustrated in Fig. 4 for docking and ensemble receptor docking. Using several structures of the same biological target, we screen the dataset with several docking programs, apply the MS protocol and use consensus scoring applying the ECR strategy. This protocol can lead to better results because it can eliminate bias towards the choice of a bad target structure or the use of a program that presents poor results for the system. The main advantages of ECR are as follows: (i) inclusion of protein target flexibility, with MS presenting more reliable results; (ii) avoidance of the problems of bias resulting from the score scales, offset or units because it is based on the rank; and (iii) ability to assign good scores to molecules that are well ranked by several docking programs, without the need to be well ranked by all of the docking programs, because it works as a conditional “or” (see Supplementary Table S1). In addition, ECR (unlike RbV) is almost independent of the threshold used and the error due to data management is negligible. In summary, these advantages allow ECR to perform better than traditional consensus strategies. Moreover, ECR requires less computational resources than other consensus strategies that have recently been proposed based on machine learning^34^, and it is less subject to bias due to the training-set dependencies of these methods.

## Conclusions

By performing docking-based virtual screening studies on several molecular systems and using seven scoring functions, in this work, we show that some consensus scoring methodologies avoid the system-bias effects that are typically found in individual docking programs, such as parameter training or overfitting. Moreover, we find that consensus strategies provide an overall better performance than the average of individual docking programs.

We highlight that rank-based consensus methodologies have several advantages compared to score-based strategies, stemming from scores having variable units, scales and offsets, which may significantly hinder combining results from individual structures and docking programs. Rank-based consensus metrics are not limited by score scales or offsets and may exhibit a better performance, as can be seen from the EFs, EPs and ROC-AUCs presented here.

We introduce a novel ranked-based consensus metric, Exponential Consensus Rank (ECR), which uses an exponential distribution to combine the ranks from individual docking programs. This metric has the advantage that its results are basically independent of the *σ* parameter, in contrast to the ‘rank-by-vote’ (RbV) methodology, which highly depends on the vote threshold. In addition, we find that ECR presents smaller errors than RbV because the assignment of the same ECR score to a molecule is less common than the assignment of the same number of votes.

Importantly, our work shows that the novel ECR metric outperforms other consensus strategies, which is independent of the *σ* parameter, the docking programs, or the system. Moreover, coupling our ECR metric with the merging and shrinking approach for receptor ensemble docking ensures better results with respect to the use of individual structures. These facts allow us to postulate the protocol shown in Fig. 4 to ensure the best results in a virtual screening process for docking and ensemble receptor docking.

## Methods

### Benchmark systems

We chose four benchmark targets to assess docking programs and consensus strategies: cyclin-dependent kinase 2 (CDK2), estrogen receptor *α* (ESR1), carbonic anhydrase 2 (CAH2), and *β*_2_ adrenergic receptor (ADRB2). These systems correspond to diverse protein families: protein kinases, nuclear receptors, carbonic anhydrases, and G protein-coupled receptors, respectively. To include the effect of the target’s flexibility, two crystal structures with differences near the active site were chosen for each system. We also selected different decoy/ligand datasets for each system, which contain known ligands and decoy (non-binder) molecules; to avoid artificial enrichment, the latter were chosen to ensure physicochemical similarity to the ligands but structural dissimilarity^59^ (see below). The diversity of these benchmark sets leads to an unbiased assessment of the protocols.

Detailed descriptions of the preparation for the structures of each system, and the molecule datasets are described below:

#### Cyclin-dependent kinase 2 (CDK2)

Two structures from the Protein Data Bank (PDB), codes 1FVV and 4KD1, were selected because they have acceptable crystallographic resolutions (2.8 Å and 1.7 Å, respectively), and show structural differences near the active site. The structures were protonated using Open Babel^60^. For this system, we used the Directory of Useful Decoys (DUD)^37^, which contains 72 ligands and 2074 decoys. In the case where more than one tautomer for a given molecule is present in the dataset, the docking pose with the best score for each program was chosen, resulting in only 50 ligands and 1779 decoys.

#### Estrogen receptor α (ESR1)

The 1XP9 and 3ERT structures (PDB codes) were selected. The NRLiSt BDB^61^, a dataset of ligands, decoys and targets for ESR1, was used. This dataset contains ligands with two types of activities, agonists and antagonists. We chose the dataset of antagonist compounds because both structures are bound to antagonists in the PDB. The antagonist dataset contains 133 ligands and 6555 decoys. Ligands with non-parameterized types of atoms for AutoDock were discarded, so finally, only 126 ligands remained. The protonation states of the target, ligands and decoys were preserved as they were in the dataset^61^.

#### β_2_ adrenergic receptor (ADRB2)

We selected the 3PDS and 4LDO PDB structures. The ligands/decoys dataset used for this system was the GPCR Decoy Database/GPCR Ligand Library (GDD/GLL)^62^. We chose the agonist dataset because the structures selected are in their active form^63,64^. This dataset has 206 ligands and 8034 decoys. The protonation state of the molecules (corresponding to *pH* = 7.4) was conserved.

#### Carbonic anhydrase 2 (CAH2)

The 1BCD and 4PQ7 structures (PDB codes) for CAH2 were selected. The structures were protonated at pH 7 using the propka3.1 server^65,66^ to predict the protonation states of the titrable residues. Additionally, MolProbity^67^ and visual inspection were used to determine the possible flips of the HIS, GLN and ASN side chains. The hydrogen atom positions were assigned using ICM, and protein and ligand polar hydrogens within 6 Å of the ligand were re-optimized using a Monte Carlo-based energy optimization^68^. The Directory of Useful Decoys, Enhanced (DUD-E) dataset^59^ was used for this enzyme. The original dataset was reduced to 7987 compounds that were randomly selected. The ligand and decoy proportions were kept as in the dataset. When more than one tautomer for a given molecule was present in the dataset, the tautomer with the best score for each program was chosen.

### Docking calculations

For molecular docking the following six programs were used: AutoDock^52^, ICM^48^, LeDock^53^, rDock^54^, AutoDock Vina^49^ and Smina^55^. These programs are characterized by having different search algorithms and scoring functions, as described below.

*AutoDock* is the most commonly used docking program, possibly, because it is one of the oldest open-source academic programs. AutoDock has been reported to accurately predict docking poses^16^. AutoDock uses a Lamarckian genetic algorithm and an empirical free energy force field scoring function^52^.

*ICM* performs rigid-receptor:flexible-ligand docking, where the receptor is represented by six potential energy maps and the docked molecule is considered flexible within the energy field of the receptor and subjected to a global energy minimization protocol that consists of Monte-Carlo sampling with local energy minimization of the differentiable variables^48^. The lowest energy pose for each molecule is assigned an empirical score according to its fit within the binding site^69^.

*LeDock* uses a combination of simulated annealing and evolutionary optimization for the ligand pose. Physics and knowledge-based hybrid scoring schemes derived from prospective virtual screening campaigns are used^53^.

*rDock* uses a docking protocol with three stages for conformational sampling: a genetic algorithm search (GA1, GA2, GA3), followed by a low temperature Monte Carlo (MC) and a Simplex minimization stage. rDock has a scoring function constructed from the sum of several pseudo-energy scoring functions^54^.

*AutoDock Vina* employs an iterated local search global optimizer. The main advantage of this program is the speed of the calculations, which facilitates virtual screening campaigns while maintaining a good scoring power^47,50^.

*Smina* is a fork of AutoDock Vina that uses a scoring function called Vinardo^70^ with enhanced features based on AutoDock Vina.

The parameters of the box size remained almost the same for all programs: 20 × 20 × 20 Å for CDK2, ADRB2 and CAH2; and 25 × 25 × 25 Å for ESR1. After aligning the two structures for each system, the box centres were defined by the coordinates shown in Supplementary Table S12. For rDock, the box was automatically built using the ligand-based method^54^, in which the free space that can be occupied by a ligand in the binding pocket is taken as the volume of docking. For this purpose, the ligands in the structures, mentioned in the Benchmark systems sub-section, were used as references. The number of requested poses was 50 for all programs except for ICM, where the number of poses is variable and the lowest-energy pose is scored. The specific details of the search parameters for each algorithm can be found in the Supplementary Files section in the Supplementary Information. These programs were chosen based on reports from the literature^44,47,50^. Each program took between 1 and 25 minutes per molecule, with AutoDock being the most time consuming (see Supplementary Table S13).

In addition, we used the random scoring function (RSF), a synthetic scoring function that assigns random scores to each molecule derived from a Gaussian distribution. We used the RSF to evaluate the ability of each consensus strategy when there was a scoring function that could not differentiate between ligands and decoys in any system.

### Merging and shrinking strategy

To consider the flexibility of protein targets, the merging and shrinking procedure (MS)^22,58^ was used. In this procedure, the docking results of the individual structures are merged, taking only the best rank (or score) for each molecule. These results are used to obtain a single rank, or score, for each molecule in receptor ensemble docking. We note that to apply the score-based consensus strategies, with MS, it is necessary to perform the MS procedure using the score. The consensus results could differ when using the rank or the score, making its selection a key issue for the virtual screening processes (see the Results section).

### Consensus scoring and ranking

The results from the individual docking programs were combined using several consensus approaches. ECR was validated by comparing its results to those obtained from the consensus strategies presented below.

#### Rank by rank (RbR)

The molecular candidates are ranked using the average rank over all of the docking programs^40^. Let ${r}_{i}^{j}$ be the rank of molecule *i* for the *j* docking program; then, the final rank of molecule *i* is given by

3 $$Rb{R}_{i}=\frac{1}{n}\,\sum _{j}\,{r}_{i}^{j},$$

where *n* is the total number of docking programs.

#### Rank by vote (RbV)

In this strategy, each molecule receives a vote if it is ranked in the top *x*% of the results for a certain docking program^40^. The final score for each molecule is given by the sum of votes obtained from all of the programs. This number can range from zero to the total number of scoring functions under consideration. All of the candidates are ranked according to their final votes. The outcome consists of the list of molecules with votes, where many molecules can have the same number of votes (*e*.*g*., for the scoring functions used in the Results section, the range is only between zero and seven). To properly estimate the performance metrics of molecules that have the same score (for ECR) or number of votes (for RbV), these molecules should be randomly shuffled several times, and the average and standard deviation of the EFs and EPs should be computed.

#### Rank by number (RbN)

The score of each molecule corresponds to the average score of the molecule considering all of the scoring functions,

4 $$Rb{N}_{i}=\frac{1}{n}\,\sum _{j}\,{s}_{i}^{j},$$

where ${s}_{i}^{j}$ is the score of molecule *i* for docking program *j*, and *n* is the total number of docking programs^40^.

#### Average of auto-scaled scores (AASS)

This method is similar to RbN but it first normalizes each score between 0 and 1 and is attempt to avoid some of the issues encountered when comparing scores from different docking programs due to the score scale or offset^36^. For each docking program *j*, the score of each molecule *i* is scaled to a number between 0 and 1 using the minimum and maximum scores for each program (${s}_{{\min }}^{j}$ and ${s}_{{\max }}^{j}$, respectively). The final score of each molecule is given by the average of all the normalized scores,

5 $$AAS{S}_{i}=\frac{1}{n}\,\sum _{j}\,\frac{{s}_{i}^{j}-{s}_{{\min }}^{j}}{{s}_{{\max }}^{j}-{s}_{{\min }}^{j}}.$$

#### Z-score

The molecule score (${s}_{i}^{j}$) is scaled using the average (*μ*^*j*^) and standard deviation (*σ*^*j*^) of the scores of all of the molecules for each docking program *j*^41^. The final score is the average of the scaled-score among all of the scoring functions,

6 $$Z-scor{e}_{i}=\frac{1}{n}\sum _{j}\frac{{s}_{i}^{j}-{\mu }^{j}}{{\sigma }^{j}}\mathrm{\ .}$$

### Metric validation

#### Enrichment Factor (EFx%)

The EF measures the enrichment of active compounds in a molecular dataset given a specific percentage of the dataset (threshold). The EF is the ratio between ligands (hits) found using a certain threshold *x*% (*Hits*^*x*%^) and the number of compounds at that threshold *N*^*x*%^ normalized by the ratio between the hits contained in the entire dataset (*Hits*^100%^) and the total number of compounds *N*^100%^,

7 $$EFx=\frac{Hit{s}^{x \% }}{{N}^{x \% }}\times \frac{{N}^{100 \% }}{Hit{s}^{100 \% }}.$$

#### Enrichment Plot (EP)

The percentage of ligands recovered as a function of the top x%-ranked compounds based on the docking and consensus scores^71^. The EP allows the identification of which method performs better based on the percentage of the screened dataset.

#### Receiver Operating Characteristics (ROC) Curve and its area under the curve (ROC-AUC)

The ROC curve indicates the ability of a program to distinguish between ligands and decoys^72^. The ROC curve is created by plotting the true positive rate (*TPR*), or *sensitivity* (Eq. 8), against the false positive rate (*FPR*), or 1 – *specificity* (Eq. 9), at several thresholds,

8 $$TPR=\frac{TP}{TP+FN},$$

and

9 $$FPR=\frac{FP}{FP+TN},$$

where TP, FP, TN and FN are the true positives, false positives, true negatives and false negatives, respectively, at a specific threshold.

An important parameter that can be obtained from ROC curves is the ROC-AUC. The ROC-AUC allows us to observe the prediction capacity of a method; in this case, it accounts for the ability of each docking program to differentiate between a ligand and a decoy. A ROC-AUC greater than 0.5 indicates that the method has a better predictive ability than a random one. The better the predictive ability of the method, the closer the ROC-AUC is to 1.

It is important to mention that EPs and ROC curves can change due to the final list sorting of RbV and ECR consensus strategies. As mentioned previously for RbV, this is because many molecules can have the same final number of votes or score. For this reason, it is necessary to calculate the error bars over the plots resulting from these scoring functions (see above and the Supplementary information).

## Supplementary information

Supplementary Information
