# Supplementary material for: Exponential consensus ranking improves the outcome in docking and receptor ensemble docking
Source: Sci Rep. 2019 Mar 26;9:5142. doi: 10.1038/s41598-019-41594-3 (PMC6435795; doi:10.1038/s41598-019-41594-3)
Supplement: Supplementary file 1 — Supplementary Information [file 41598_2019_41594_MOESM1_ESM.pdf]

Supplementary information: Exponential consensus ranking improves the outcome in docking and receptor ensemble docking

Karen Palacio-Rodríguez[1], **Isaias Lans**[1], **Claudio N. Cavasotto**[2,3,4,*], **and Pilar Cossio**[1,5,*]

[1]Biophysics of Tropical Diseases Max Planck Tandem Group, University of Antioquia, Medellín, Colombia
[2]Computational Drug Design and Drug Discovery Informatics Laboratory, Translational Medicine Research Institute (IIMT), CONICET-Universidad Austral, Pilar-Derqui, Buenos Aires, Argentina
[3]Facultad de Ciencias Biomédicas, Universidad Austral, Pilar-Derqui, Buenos Aires, Argentina
[4]Facultad de Ingeniería, Universidad Austral, Pilar-Derqui, Buenos Aires, Argentina
[5]Department of Theoretical Biophysics, Max Planck Institute of Biophysics, 60438 Frankfurt am Main, Germany
[*]cnc@cavasotto-lab.net; pilar.cossio@biophys.mpg.de

Supplementary Tables

Molecules	Rank Program 1	Rank Program 2	RbR	ECR (σ=10)
◇	4	91	47.5	$\frac{e^{-\frac{4}{\sigma}}}{\sigma} + \frac{e^{-\frac{91}{\sigma}}}{\sigma} = 0.067$
✧	7	15	11	$\frac{e^{-\frac{7}{\sigma}}}{\sigma} + \frac{e^{-\frac{15}{\sigma}}}{\sigma} = 0.072$
○	48	46	47	$\frac{e^{-\frac{48}{\sigma}}}{\sigma} + \frac{e^{-\frac{46}{\sigma}}}{\sigma} = 0.002$

Table S1. Example of how the exponential consensus ranking (ECR) works. The table presents some synthetic results for three molecules (diamond, star and circle) from two different programs, and exemplifies cases that may occur when applying consensus approaches. Most consensus strategies use the average of the results from the different programs, as does the rank-by-rank (RbR) strategy. We compare these results to the ECR strategy using a $\sigma = 10$. The star molecule has a high rank for both programs, and both the RbR and ECR strategies give this molecule a good score. However, for the diamond molecule, which has a very good rank for one program but a poor rank for the other, the strategies perform differently. The RbR ranks the diamond poorly because of the averaging, and gives it a worse rank than a faulty-performing molecule (*e.g.,* the circle). Whereas, the ECR ranks it much better. By using a sum of exponential functions, the ECR acts as conditional *"or"* that allows to give more weight to the molecules that have been well ranked by at least one program but not necessarily by all.

System	CDK2		ESR1		ADRB2		CAH2	
Structure	4KD1	1FVV	1XP9	3ERT	3PDS	4LDO	1BCD	4PQ7
AutoDock	0.558	0.701	0.778	0.779	0.630	0.648	0.605	0.548
ICM	0.680	0.753	0.840	0.801	0.794	0.855	0.679	0.691
LeDock	0.729	0.745	0.705	0.727	0.607	0.690	0.763	0.762
rDock	0.602	0.729	0.861	0.896	0.802	0.812	0.843	0.834
Smina	0.572	0.630	0.764	0.792	0.758	0.732	0.708	0.745
AutoDock Vina	0.520	0.638	0.792	0.809	0.614	0.651	0.642	0.645
RSF	0.504	0.505	0.502	0.501	0.504	0.506	0.501	0.504
AASS	0.640	0.729	0.747	0.766	0.674	0.704	0.761	0.767
RbN	0.594	0.723	0.868	0.855	0.772	0.843	0.727	0.731
Z-score	0.649	0.731	0.821	0.857	0.762	0.797	0.794	0.801
RbR	0.651	0.729	0.848	0.870	0.779	0.810	0.787	0.788
RbV	0.725	0.780	0.797	0.798	0.692	0.765	0.659	0.670
ECR	0.695	0.777	0.846	0.858	0.818	0.848	0.779	0.790

Table S2. ROC-AUC for all individual programs for each structure. Normalized color code for each structure of each system, from worst (green) to best (yellow). The RbV and ECR strategies were calculated for a threshold equal to the 5% of the dataset.

System	CDK2		ESR1		ADRB2		CAH2	
Structure	4KD1	1FVV	1XP9	3ERT	3PDS	4LDO	1BCD	4PQ7
AutoDock	4.0	18.0	20.5	22.8	6.3	8.3	0.5	2.0
ICM	8.0	22.0	29.9	26.0	13.6	25.3	1.0	3.4
LeDock	8.0	22.0	5.5	14.2	3.9	2.4	9.8	10.2
rDock	6.0	20.0	25.2	28.3	10.2	9.7	5.4	12.2
Smina	8.0	10.0	26.0	25.2	3.4	1.5	2.9	7.3
AutoDock Vina	2.0	6.0	12.6	12.6	4.4	2.9	3.9	1.0
RSF	2.0	0.0	1.6	0.8	0.0	1.0	2.0	1.0
AASS	12.0	28.0	15.0	15.0	2.9	5.3	10.7	9.3
RbN	12.0	22.0	33.1	27.6	9.7	19.9	7.3	9.3
Z-score	12.0	28.0	29.9	33.1	11.7	9.7	14.2	12.2
RbR	12.0	22.0	25.2	26.0	11.7	10.7	12.2	8.3
RbV	9.4	26.2	30.0	32.6	10.9	10.2	10.4	13.6
ECR	12.0	28.0	33.9	35.4	12.1	11.7	12.2	13.2

Table S3. Enrichment Factor at 1% (EF1) for all individual programs with each structure. Normalized color code for each structure of each system, from worst (green) to best (yellow). The RbV and ECR strategies were calculated for a threshold equal to the 5% of the dataset.

System	CDK2		ESR1		ADRB2		CAH2	
Structure	4KD1	1FVV	1XP9	3ERT	3PDS	4LDO	1BCD	4PQ7
AutoDock	2.8	7.2	7.1	7.2	2.7	3.3	2.3	2.5
ICM	4.8	7.2	9.9	8.5	7.7	10.3	2.1	2.2
LeDock	5.2	8.4	6.1	6.8	2.8	3.9	5.8	5.6
rDock	2.4	7.6	9.0	10.4	5.0	6.2	5.9	6.5
Smina	2.8	4.4	8.4	8.2	4.4	2.9	3.1	5.1
AutoDock Vina	1.6	5.2	7.2	7.2	3.5	2.3	3.3	2.4
RSF	2.0	1.2	1.0	0.8	0.5	0.9	1.3	0.9
AASS	4.0	9.2	6.3	6.5	3.2	3.9	5.4	5.6
RbN	4.8	6.0	10.5	9.3	6.1	9.7	3.9	3.7
Z-score	3.6	9.6	9.5	10.2	6.3	7.2	6.1	6.5
RbR	3.6	8.8	9.5	10.4	6.1	7.5	5.8	5.6
RbV	4.8	9.5	9.9	10.4	5.9	6.9	5.8	6.0
ECR	4.0	9.6	10.2	11.0	6.6	8.3	6.1	7.4

Table S4. EF5 for all individual programs with each structure. Normalized color code for each structure of each system, from worst (green) to best (yellow). The RbV and ECR strategies were calculated for a threshold equal to the 5% of the dataset.

System	CDK2		ESR1		ADRB2		CAH2	
Structure	4KD1	1FVV	1XP9	3ERT	3PDS	4LDO	1BCD	4PQ7
AutoDock	3.0	13.0	14.6	13.8	3.6	5.8	0.7	2.2
LeDock	10.0	14.0	6.7	9.8	2.2	3.6	8.1	7.1
rDock	4.0	16.0	16.5	19.7	6.6	6.8	5.1	8.8
Smina	5.0	6.0	15.4	15.0	4.4	1.2	3.4	5.6
AutoDock Vina	2.0	7.0	9.8	9.5	4.6	2.2	3.2	2.0
RSF	2.0	2.0	0.8	0.4	0.0	1.0	1.2	0.7
AASS	6.0	17.0	10.2	9.8	1.9	6.4	7.8	6.3
RbN	4.0	6.0	12.2	9.1	3.9	5.1	5.4	3.9
Z-score	6.0	17.0	16.5	19.7	4.9	5.6	9.3	8.8
RbR	6.0	14.0	13.0	11.4	4.9	7.0	7.8	6.8
RbV	5.3	17.5	18.1	20.2	6.3	5.5	8.0	9.4
ECR	6.0	19.0	20.1	22.4	7.3	7.0	8.1	9.5

Table S5. EF2 for all individual programs with each structure taking out the ICM program. Normalized color code for each structure of each system, from worst (green) to best (yellow). The RbV and ECR strategies were calculated for a threshold equal to the 5% of the dataset.

System	CDK2		ESR1		ADRB2		CAH2	
Structure	4KD1	1FVV	1XP9	3ERT	3PDS	4LDO	1BCD	4PQ7
$RbV_{1\%}$	5.2	15.3	19.0	20.0	7.0	8.2	4.0	5.9
$RbV_{2\%}$	7.6	18.4	20.3	22.9	8.3	8.4	5.3	6.8
$RbV_{5\%}$	6.8	19.2	19.9	21.1	9.2	9.5	8.4	9.6
$RbV_{10\%}$	6.2	16.3	18.5	18.9	8.8	8.3	9.3	9.8
$RbV_{25\%}$	6.6	13.9	12.6	14.7	5.5	7.8	7.7	7.2
$RbV_{50\%}$	3.5	6.4	5.9	5.6	4.0	5.2	4.4	3.8
$ECR_{1\%}$	8.0	19.0	22.8	23.6	8.0	9.5	4.9	7.1
$ECR_{2\%}$	7.0	21.0	22.1	24.8	10.0	9.7	7.3	8.1
$ECR_{5\%}$	6.0	21.0	21.7	22.8	11.9	10.4	8.3	9.0
$ECR_{10\%}$	6.0	19.0	22.1	22.1	10.7	10.7	9.8	10.0
$ECR_{25\%}$	6.0	20.0	21.3	20.9	9.5	11.4	10.0	9.8
$ECR_{50\%}$	6.0	19.0	20.5	20.5	9.7	10.7	9.8	8.8

Table S6. The RbV and ECR have an external parameter (vote-threshold and σ, respectively) that controls the number of molecules to be taken for the consensus. In this Table, we show the EF2 of the RbV and ECR consensus strategies for vote-thresholds and σ parameters equal to 1%, 2%, 5%, 10%, 25% and 50% of the dataset. Interestingly, we find that while the RbV highly depends on the vote-threshold, the results from the ECR are almost independent over a wide range of σ. The almost independence of the ECR as a function of the σ parameter gives it an advantage over the RbV strategy. Therefore, it is possible to apply ECR without worrying about losing performance. Normalized color code for each structure of each system from worst (green) to best (yellow).

System	CDK2		ESR1		ADRB2		CAH2	
Merging and Shrinking	Score	Rank	Score	Rank	Score	Rank	Score	Rank
AutoDock	0.680	0.675	0.777	0.778	0.653	0.651	0.566	0.580
ICM	0.768	0.787	0.853	0.853	0.885	0.892	0.715	0.716
LeDock	0.775	0.783	0.714	0.719	0.624	0.655	0.757	0.757
rDock	0.720	0.719	0.867	0.885	0.820	0.820	0.834	0.834
Smina	0.638	0.644	0.768	0.787	0.750	0.753	0.727	0.716
AutoDock Vina	0.638	0.644	0.800	0.805	0.611	0.619	0.641	0.643
RSF	0.501	0.501	0.501	0.501	0.492	0.492	0.487	0.487
AASS	0.736		0.810		0.720		0.773	
RbN	0.730		0.871		0.846		0.744	
Z-score	0.739		0.855		0.799		0.796	
RbR		0.735		0.859		0.803		0.783
RbV		0.819		0.796		0.731		0.651
ECR		0.802		0.858		0.856		0.785

Table S7. ROC-AUC for the merging and shrinking (MS) strategy using each individual program and the consensus strategies. The MS procedure uses the best score or the best rank depending on the consensus strategy employed. Normalized color code for each system, from worst (green) to best (yellow). The RbV and ECR strategies were calculated for a threshold equal to the 5% of the dataset.

System	CDK2		ESR1		ADRB2		CAH2	
Merging and Shrinking	Score	Rank	Score	Rank	Score	Rank	Score	Rank
AutoDock	16.0	14.0	21.3	22.1	7.3	6.3	1.5	1.5
ICM	16.0	18.0	30.7	30.7	20.9	18.9	2.4	2.0
LeDock	24.0	20.0	9.5	11.0	3.9	2.9	11.2	10.7
rDock	14.0	12.0	28.3	29.1	11.2	10.7	7.8	7.8
Smina	8.0	6.0	26.8	26.8	1.5	1.9	6.3	5.9
AutoDock Vina	6.0	4.0	12.6	14.2	4.4	4.4	1.5	2.0
RSF	2.0	2.0	0.8	0.8	0.0	0.0	0.5	1.0
AASS	26.0		26.8		4.3		11.7	
RbN	14.0		35.4		17.5		10.2	
Z-score	26.0		33.9		10.7		12.2	
RbR		20.0		32.3		10.2		11.7
RbV		28.3		33.9		12.1		13.1
ECR		28.0		41.0		13.6		11.7

Table S8. EF1 for the MS strategy using each individual program and the consensus strategies. The MS procedure uses the best score or the best rank depending on the consensus strategy employed. Normalized color code for each system, from worst (green) to best (yellow). The RbV and ECR strategies were calculated for a threshold equal to the 5% of the dataset.

System	CDK2		ESR1		ADRB2		CAH2	
Merging and Shrinking	Score	Rank	Score	Rank	Score	Rank	Score	Rank
AutoDock	6.8	6.8	7.2	7.2	3.2	3.3	2.5	2.5
ICM	8.8	8.4	9.7	10.4	10.8	10.7	2.2	2.1
LeDock	8.0	8.4	6.6	6.8	2.5	3.1	5.4	5.8
rDock	6.8	6.8	9.4	10.4	5.8	5.8	5.5	5.5
Smina	4.4	4.0	8.2	8.4	3.2	3.5	4.4	4.1
AutoDock Vina	5.2	5.2	7.2	8.0	3.0	3.1	2.7	3.2
RSF	2.4	2.0	0.6	0.6	0.4	0.4	1.0	1.0
AASS	10.0		9.3		4.2		5.9	
RbN	7.6		10.7		8.3		5.2	
Z-score	10.0		10.9		7.2		6.6	
RbR		8.4		10.2		7.2		5.9
RbV		9.7		10.4		7.2		5.5
ECR		10.8		11.0		7.6		6.3

Table S9. EF5 for MS strategy using each individual program and the consensus strategies. The MS procedure uses the best score or the best rank depending on the consensus strategy employed. Normalized color code for each system, from worst (green) to best (yellow). The RbV and ECR strategies were calculated for a threshold equal to the 5% of the dataset.

System	CDK2		ESR1		ADRB2		CAH2	
Merging and Shrinking	Score	Rank	Score	Rank	Score	Rank	Score	Rank
AutoDock	12.0	11.0	14.6	14.6	5.3	4.9	1.2	1.2
LeDock	15.0	15.0	8.7	9.1	2.2	2.7	7.8	7.6
rDock	11.0	11.0	17.7	19.3	8.0	8.0	7.3	8.1
Smina	6.0	7.0	15.8	15.4	1.9	2.4	4.4	4.4
AutoDock Vina	7.0	4.0	9.8	11.0	3.4	3.4	2.0	2.4
RSF	2.0	1.0	1.2	1.2	0.5	0.5	1.5	1.5
AASS	15.0		14.6		2.9		8.1	
RbN	8.0		13.4		5.1		6.3	
Z-score	15.0		18.9		4.6		8.8	
RbR		12.0		15.0		5.8		7.1
RbV		14.7		20.0		6.6		8.9
ECR		17.0		20.9		8.0		9.5

Table S10. EF2 for the MS strategy for the individual programs and consensus strategies taking out the ICM program. Normalized color code for each system, from worst (green) to best (yellow). The RbV and ECR strategies were calculated for a threshold equal to the 5% of the dataset.

System	CDK2	ESR1	ADRB2	CAH2
AutoDock	1FVV	1XP9	4LDO	4PQ7
ICM	1FVV	1XP9	4LDO	4PQ7
LeDock	1FVV	3ERT	4LDO	1BCD
rDock	1FVV	3ERT	4LDO	4PQ7
Smina	1FVV	1XP9	3PDS	4PQ7
AutoDock Vina	1FVV	1XP9	3PDS	1BCD
RSF	1FVV	1XP9	4LDO	1BCD

Table S11. Best structure for each system and program. We select the structure with higher EF2 as the best structure.

System	Reference	Center x	Center y	Center z
CDK2	4KD1	52.4	82.9	21.5
ESR1	3ERT	30.7	-1.0	27.1
ADRB2	4LDO	-1.6	-12.7	-48.3
CAH2	1BCD	-1.9	7.2	13.7

Table S12. Coordinates of box centers for each system. The second structure of each system (used in this study) was aligned to the reference structure.

System	CDK2	ESR1	ADRB2	CAH2
AutoDock	22	18	25	20
ICM	0.5	0.5	0.5	0.5
LeDock	2.5	2	4	3
rDock	6	5	9	6.5
Smina	1.5	1	2	1.5
AutoDock Vina	1.5	1	2	1.5

Table S13. Average computational time per molecule on a single core. Time given in minutes.

Supplementary Figures

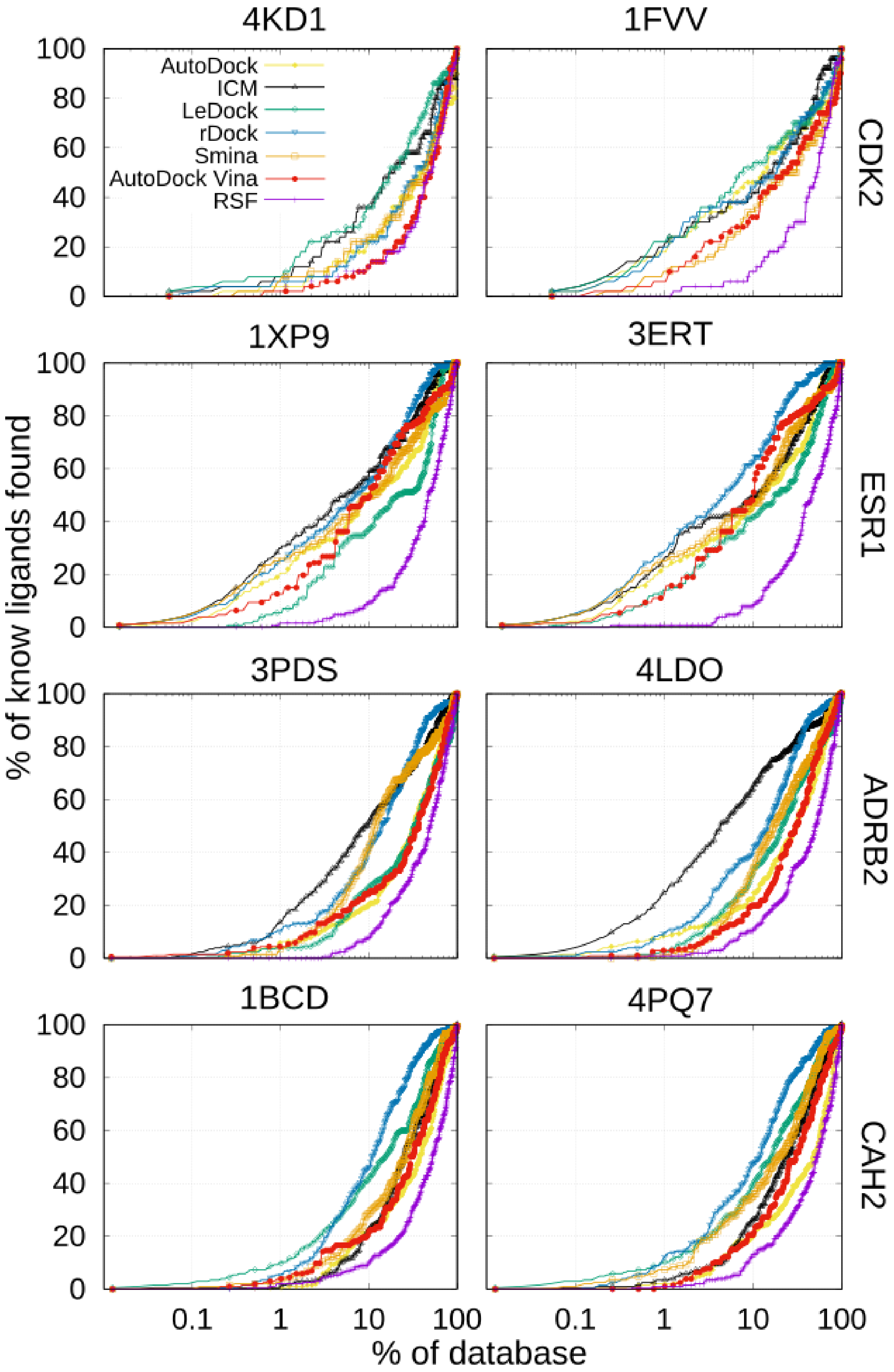

Figure S1. Enrichment plots for all individual programs before applying the MS strategy. As expected, the RSF presents the worst results because it has no ability to filter between ligands and decoys. The other programs have performances that depend on each system. ICM and rDock present generally good results. LeDock presents good results only for the CDK2 and CAH2. AutoDock presents similar results for all systems and is close to the average of the other programs. AutoDock Vina and Smina are the programs that present poorer performances for the individual structures.

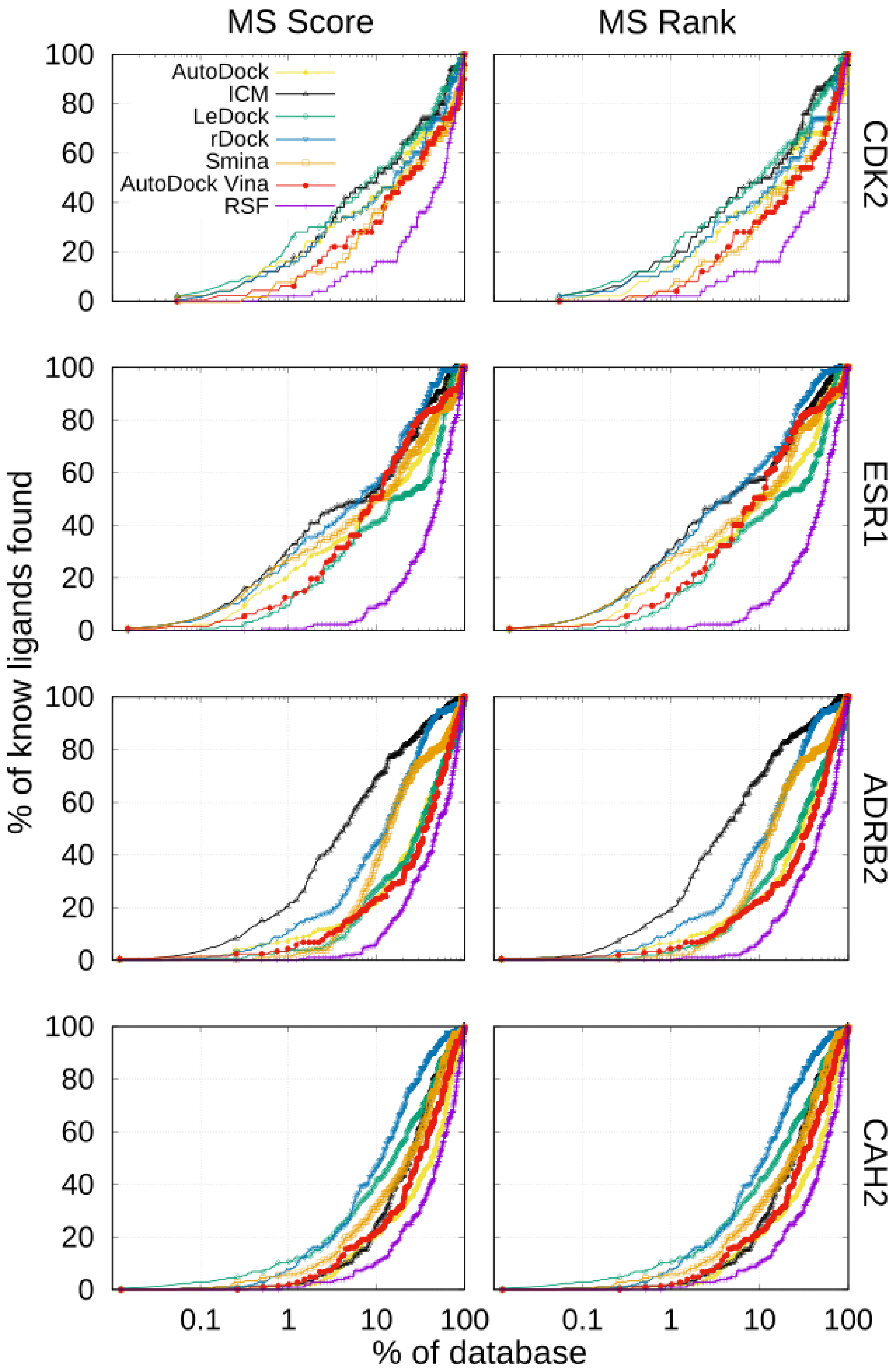

Figure S2. Enrichment plots for all individual programs after applying the MS strategy. No significant differences were observed between MS score and MS rank. In most cases, the performance of the individual programs improves with the MS.

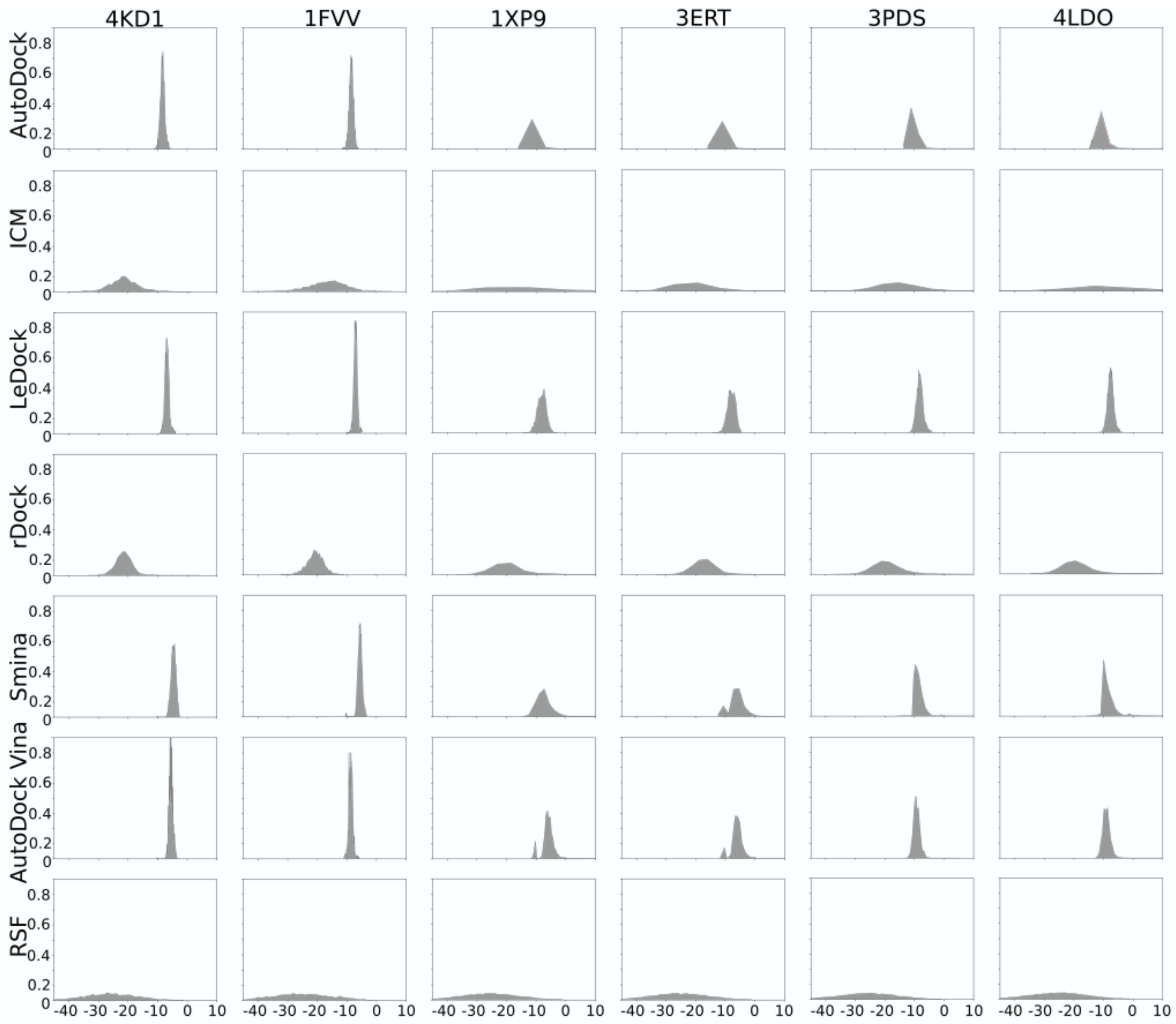

Figure S3. Distributions of scores for each docking program. The scoring functions are highly parameterized potentials that can give results (scores) in different units, scales or offsets. To understand the behavior of consensus strategies based on the score, we evaluate the distributions and compare the energy scales and offsets. For example, very negative scales can skew the average in RbN. Broad or narrow score distributions can affect the results of the AASS or the Z-score strategies.

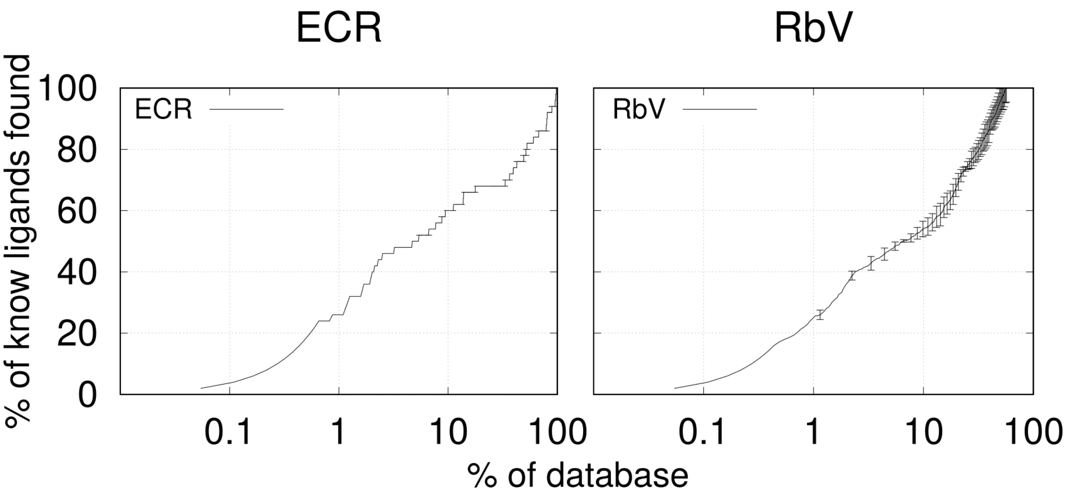

Figure S4. EP plot with errors bars for the CDK2 1FVV structure applying the ECR (left) and RbV (right) using a threshold-parameter at 5%. As mentioned in the methods, it is important to take care of the list-sorting with the RbV and ECR metrics because many molecules can have the same number of votes or ECR probability-score. We find that errors in ECR begin to occur at higher percentages of the dataset and are almost negligible, contrary to what is presented in RbV, where in any percentage of the dataset taken errors can occur due to this random sort.

Supplementary Files

- AutoDock parameter input file:

```
autodock_parameter_version 4.2          # used by autodock to validate parameter set
outlev 1                                # diagnostic output level
intelec                                 # calculate internal electrostatics
seed pid time                           # seeds for random generator
ligand_types A C HD N OA S              # atoms types in ligand
fld 1bcd_active_2.maps.fld              # grid_data_file
map 1bcd_active_2.A.map                 # atom-specific affinity map
map 1bcd_active_2.C.map                 # atom-specific affinity map
map 1bcd_active_2.HD.map                # atom-specific affinity map
map 1bcd_active_2.N.map                 # atom-specific affinity map
map 1bcd_active_2.OA.map                # atom-specific affinity map
map 1bcd_active_2.S.map                 # atom-specific affinity map
elecmap 1bcd_active_2.e.map             # electrostatics map
desolvmap 1bcd_active_2.d.map           # desolvation map
move active_2.pdbqt                     # small molecule
about 3.4491 -0.5907 1.1184             # small molecule center
tran0 random                            # initial coordinates/A or random
quaternion0 random                      # initial orientation
dihe0 random                            # initial dihedrals (relative) or random
torsdof 7                               # torsional degrees of freedom
rmstol 2.0                              # cluster_tolerance/A
extnrg 1000.0                           # external grid energy
e0max 0.0 10000                         # max initial energy; max number of retries
ga_pop_size 150                         # number of individuals in population
ga_num_evals 2500000                    # maximum number of energy evaluations
ga_num_generations 27000                # maximum number of generations
ga_elitism 1                            # number of top individuals to survive to next gener
ga_mutation_rate 0.02                   # rate of gene mutation
ga_crossover_rate 0.8                   # rate of crossover
ga_window_size 10                       #
ga_cauchy_alpha 0.0                     # Alpha parameter of Cauchy distribution
```

```
ga_cauchy_beta 1.0                    # Beta parameter Cauchy distribution
set_ga                                # set the above parameters for GA or LGA
sw_max_its 300                        # iterations of Solis & Wets local search
sw_max_succ 4                         # consecutive successes before changing rho
sw_rho 1.0                            # size of local search space to sample
sw_lb_rho 0.01                        # lower bound on rho
ls_search_freq 0.06                   # probability of performing local search on individu
set_psw1                              # set the above pseudo-Solis & Wets parameters
unbound_model bound                   # state of unbound ligand
ga_run 50                             # do this many hybrid GA-LS runs
analysis                              # perform a ranked cluster analysis
```

- ICM general docking parameters:

```
l_internalHB = yes
l_neutralAcidsMol = no
s_chargeGroups NH2 NH NT
thoroughness = 2
```

- LeDock parameter input file:

```
Receptor
1bcd.pdb
RMSD
1.0
Binding pocket
-11.5 8.5                 # xmin xmax
-3.5 16.5                 # ymin ymax
5.0 25.0                  # zmin zmax
Number of binding poses
50
Ligands list
ligand.list
END
```

- rDock parameter input file:

```
RBT_PARAMETER_FILE_V1.00
TITLE 1bcd

RECEPTOR_FILE 1bcd.mol2
RECEPTOR_FLEX 3.0

##################################################################
### CAVITY DEFINITION: REFERENCE LIGAND METHOD
##################################################################
SECTION MAPPER
    SITE_MAPPER RbtLigandSiteMapper
    REF_MOL 1bcd_ligand.sd
    RADIUS 6.0
    SMALL_SPHERE 1.0
    MIN_VOLUME 100
    MAX_CAVITIES 1
    VOL_INCR 0.0
   GRIDSTEP 0.5
```

```
END_SECTION

##################################
#CAVITY RESTRAINT PENALTY
##################################
SECTION CAVITY
    SCORING_FUNCTION RbtCavityGridSF
    WEIGHT 1.0
END_SECTION
```

- Smina parameter input file:

```
receptor = 1bcd.pdbqt
ligand = ZINC.pdbqt
center_x = -1.9
center_y = 7.2
center_z = 13.7
size_x = 20
size_y = 20
size_z = 20
out = ZINC_out.pdbqt
log = ZINC_log.txt
cpu = 1
exhaustiveness = 9
num_modes = 50
scoring = vinardo
```

- Vina parameter input file:

```
receptor = 1bcd.pdbqt
ligand = ZINC.pdbqt
center_x = -1.9
center_y = 7.2
center_z = 13.7
size_x = 20
size_y = 20
size_z = 20
out = ZINC_out.pdbqt
log = ZINC_log.txt
cpu = 1
exhaustiveness = 9
num_modes = 50
```
